# Correction to “Clinical Pharmacology Perspective on Direct‐To‐Subcutaneous Dosing of T Cell Engagers in Oncology First‐In‐Human Studies”

**DOI:** 10.1111/cts.70483

**Published:** 2026-01-22

Qiao W., Shahraz A., Vishwanathan K. and Sawant‐Basak A. Clinical pharmacology perspective on direct‐to‐subcutaneous dosing of T cell engagers in oncology first‐in‐human studies. *Clinical and Translational Science*. 2026; 19(1):e70461.

In Conflicts of Interest section, the statement of “The authors declare no conflicts of interest” was incorrect.

This should have been “W.Q. was an employee of AstraZeneca when the work was conducted. A.S., K.V., and A.S‐B are employees and shareholders of AstraZeneca.”

We apologize for this error.

